# How is occupational identity affected by the family environment of publicly funded students in local normal colleges? A moderated mediation model

**DOI:** 10.3389/fpsyg.2023.934133

**Published:** 2023-08-16

**Authors:** Jia Zhang, Yang Liu, Zhi Mao, Bei Xiao

**Affiliations:** ^1^School of Education and Psychology, Chengdu Normal University, Chengdu, Sichuan, China; ^2^Research Center of Mental Health Education, Faculty of Psychology, Southwest University, Chongqing, China; ^3^Department of Maritime, Sichuan Vocational and Technical College of Communications, Chengdu, Sichuan, China

**Keywords:** occupational identity, family environment, local publicly funded students, psychological capital, professional commitment

## Abstract

The occupational identity of local publicly funded students is associated with the stability of rural teachers. To discuss the influence mechanisms of family environment, psychological capital, and professional commitment on occupational identity, this study examined 395 local publicly funded students with the occupational identity scale, psychological capital scale, professional commitment scale compiled, and family environment scale in China. We found no significant difference in terms of sex, source of birth, only children or not, and from teachers' families or not. Junior students' occupational identity was significantly higher than that of freshmen. In addition, we found that psychological capital plays a total mediation role between family environment and occupational identity. Psychological capital explains the influence of family environment on occupational identity. While the moderating role of professional commitment on the family environment and psychological capital is not supported in this study, it positively moderates the mediation role of psychological capital and occupational identity. Overall, this study will be significant in improving the training quality of local publicly funded students.

## 1. Introduction

In recent years, most countries have been facing the problem of the loss of rural teachers. Governments and academic circles worldwide are attempting to analyze the causes of the problem and formulate policies accordingly. To explain the reasons behind the hemorrhage among qualified teachers, researchers have drawn attention to the identity definition used in the philosophy and social sciences (Beijaard et al., 2004). Specifically, occupational identity serves as a critical driving force behind teachers' self-growth and professional development, thereby determining their attitude, cognition, emotion, and behaviors toward their profession to a substantial extent (Beijaard et al., 2004; Rodrigues and Mogarro, 2019). Furthermore, the development of occupational identity represents a fluid and changeable social process that involves individual and collective perception and differentiation (Sherry, 2008). Owing to its complex and multifaceted nature, occupational identity is associated with environmental concerns, such as educational relationships and experiences and individual characteristics (Rodrigues and Mogarro, 2019). While interacting with their environment, individuals' identity formats within social contexts (Erikson, 1968), particularly for the student teachers (Beijaard et al., 2004; Beauchamp and Thomas, 2009; Pillen et al., 2013).

A previous study identified certain factors influencing the career choices of students. Meanwhile, research and empirical findings from different nations demonstrate that students' family backgrounds, abilities, personalities, and motivations are the key factors that determine their selection of teacher education majors (Savage et al., 2021). In addition, family and school environments constitute the major contextual factors that shape the study-to-work transition (Hargrove et al., 2002; Restubog et al., 2010; Garcia et al., 2012). For instance, Montmarquette et al. (2002) confirmed that the family wealth and socioeconomic status of adolescents are positively related to their educational choices. Consistent with this, students seek emotional support from their families, which leads to the development of positive behavior tendencies among them as a positive psychological capital, thus effectively enhancing their professional identity (Gang and Da-Jun, 2014). As a result, family support results in higher levels of career adaptability (Zheng et al., 2023). Noticeably, career adaptability is interrelated with the development of a strong occupational identity (Kirchknopf, 2002). Based on this, college students may experience identity diffusion in families with poorly defined boundaries; therefore, students prefer to avoid an occupational choice or commit to an option without exploration to fulfill parental expectations (Berríos-Allison, 2005).

At the same time, professional commitment is a psychological reaction to an individual's profession, which is originally shaped by fundamental education. Such a psychological reaction tends to be stable during the period of graduation; additionally, this reaction is characterized by continuous variation following graduation (Lee et al., 2000; Sibandze and Scafide, 2017). For normal students, professional commitment refers to whether they plan to work in their professional field after graduation (Hall et al., 2005). Reportedly, Chinese education students come from less privileged backgrounds in terms of socioeconomic status and academic preparation; furthermore, a significant number of these students do not intend to commit to teaching as a lifelong career (Su et al., 2001). As a result, it is critical to strengthen normal students' professional dedication (Wang et al., 2016). Therefore, improving students' professional commitment has important benefits for their academic performance and career development, which is an issue that teachers and normal colleges should consider (Su and Yue, 2020).

As a form of free education and national funding, local publicly funded students have already signed contracts with the local government before leaving their hometowns, thereby agreeing in advance to return to local primary and middle schools in the rural areas of their hometown for employment after graduation. Consequently, the role of psychological capital and professional commitment between family environment and occupational identity can improve normal students' ability under pressure and maintain an optimistic attitude toward professional study and future work (Luthans et al., 2007b), which can be intervened to enhance the psychological capital and occupational identity of students.

A total of 28 Chinese provinces (districts or cities) have implemented such policies, with nearly 40,000 teachers and students of primary and secondary schools being supplemented to rural and remote regions each year, after more than 10 years of exploration. However, after several years of implementation, it has reflected problems of immature career choices and brain drain (Su et al., 2001). Hence, in contrast to ordinary normal students, strengthening the occupational identity among publicly funded students becomes an imperative goal of this policy to carry out the smooth performance. Despite the practical and theoretical significance of occupational identity, very little research has been undertaken on the relationship between family influence and occupational identity. In line with this, it is essential to understand the factors that are perceived as significant by the local publicly funded students from the perspective of occupational identity. Thus, to explore its underlying mechanisms for enhancing vocational identity among local publicly funded students, this study addresses two major research questions.

1. What are the factors that affect the occupational identity of publicly funded students at local normal colleges in their future careers?
2. What is the association among those factors of publicly funded students at local normal colleges?

## 2. Literature review and theoretical background

### 2.1. Family environment and occupational identity

Family is the first place for teenagers to study and live. Family hard environments (i.e., family income, parent's education level, and parents' occupation) and soft environments (i.e., attitude toward education, intimate family atmosphere, and children's rearing style; Hart and Risley, 1995) closely correlate with teenagers' self-efficacy of career decision-making, career exploration behavior, and planning. Normal students' occupational identity denotes their perception and experience of the profession they will engage in, which is the psychological basis and preparation for the teaching profession, the key factor for teachers' resignation, and the most lasting source of teachers' emotions (Feng et al., 2010).

The economic, cultural, and social resources owned by different families, parents' educational attitudes toward their children, educational methods, and educational expectations exert a critical impact on their children's educational choices to varying degrees (Wen and Hongyun, 2018). The investigation of the development stage revealed that the development of teachers' occupational identities follows an internal phased track of knowledge, ideas, and behavior (Jingping, 2019). Some studies demonstrated that families' cultures could shape individual professional values and professional ideas (Mian et al., 2007). The intimacy of the family environment influences individual self-cognition and professional behavior and enhances self-efficacy (Hong, 2010). The control of the family environment can standardize behavior and morality, deepen the internalization of norms, and promote the unity of knowledge and practice (Lamote and Engels, 2010). In addition, personality qualities, such as diligence and tenacity, and behavior norms and family culture that have been developed for a long time in the process of family development and restrict family members, exert a subtle impact on students' learning consciousness. In the same vein, family background, social status, and environment exhibit a substantial impact on the future college expectations and career choices of high school seniors (Ozdemir and Hacifazlioglu, 2008). As a special group undertaking the cultivation of rural education teachers, through practice in recent years, publicly funded normal students have shown great randomness and uncertainty in policy cognition, voluntary reporting, teaching motivation, and professional preferences and are more susceptible to the influence of the family environment (Zhao and Qi, 2011). Accordingly, the following is hypothesized:

Hypothesis 1: Family environment positively affects the occupational identity of publicly funded normal students.

### 2.2. The mediating role of psychological capital

Psychological capital is an individual's psychological potential and positive advantage that can encourage employees to complete their in-role behavior and display organizational citizenship behavior aimed at helping others (Luthans et al., 2007b). Meanwhile, some studies defined the four positive psychological states of self-confidence, hope, optimism, and tenacity as the core concept of psychological capital (Luthans and Youssef, 2004). Cole claimed that psychological capital could mediate other variables to affect employee behavior. He took unemployed employees as the research object and found that psychological capital plays an intermediary role in correlating subjective satisfaction after unemployment and reemployment behavior (Cole and Wellbeing, 2006). Renn and Vandenberg (1995) established that psychological factors play an intermediary role in the influence of personal factors (such as personal ability and effort level). Although no research has been conducted on the role of psychological capital in the family environment on normal students' occupational identities in pedagogy, studies have confirmed that family intimacy exerts a positive predictive effect on college students' psychological capital (Zhan and Li, 2019). A good family environment can not only directly affect the individual's psychological capital but also make the individual more aware of the support from friends and others outside the family (Wang and Youzhi, 2010). Besides, some studies confirmed that professional identity and psychological capital as intermediary effects could significantly enhance college students' employability (Gao et al., 2017). College teachers' professional honor can markedly predict teachers' professional success and professional satisfaction through psychological capital intermediary variables (Sheikh and Aghaz, 2019); this provides a certain basis for the intermediary role of psychological capital in this study. Individuals living in a good family atmosphere can feel love and support. The family environment promotes the improvement of their psychological capital by influencing their optimistic and positive personality traits, which might affect their behavior, attitude, and choices. Accordingly, the following is hypothesized:

Hypothesis 2: Psychological capital plays an intermediary role in the influence of family environment on occupational identity.

### 2.3. Moderating role of professional commitment

The concept of professional commitment stems from organizational commitment and professional commitment. The mature three-factor model involves emotional commitment, normative commitment, and continuing commitment (Bagraim, 2003). Accordingly, professional commitment refers to a commitment to professional objectives, beliefs, values, and willingness to continue in a specific profession (Teng et al., 2009). Individuals with a high level of professional commitment put extra emphasis and priority on their profession as well as their peers in the professional community (Frost et al., 2016). The career of college students is professional learning. The commitment to professional learning reflects the positive attitude and behavior of college students' recognition, love, and willingness to make corresponding efforts and exhibit good behavior. Reportedly, the initial teaching commitment of pre-service teachers after completing the learning and training during teacher education closely correlates with whether they choose the teaching profession in the future. Besides, the 1st years of teaching constitute the entry phase to the profession and are perceived as a phase of critical importance for ascertaining the professional identity of a new teacher (Vaitzman and Izhak, 2021).

Occupational identity is primarily reflected in individual value evaluations or judgments on different characteristics of teachers' professions (Flores and Day, 2006). Professional commitment reflects the individual's psychological or emotional connection to the teacher's career (Chan et al., 2008). Identity is based on individual emotional cognition, and stable psychological or emotional connection is reflected in the value judgment of professional attitude (Sheikh and Aghaz, 2019). According to resource conservation theory, individuals can use their resources to handle stress and meet the needs of the environment to attain positive results. In terms of the mechanism of professional commitment on normal students' occupational identities, studies have established that college students' professional commitment significantly correlates with learning efficacy. In a study, the learning efficacy of college students in the high-commitment group was markedly higher than that in the low-commitment group (Yang et al., 2009). Moreover, a significant positive correlation was found between emotional commitment, ideal commitment, and self-efficacy (Feng et al., 2012). The achievement motivation of college students also correlates with professional commitment. College students with low professional commitment levels are more likely not to pursue a relevant job after graduation, which not only wastes educational resources but also reduces their enthusiasm for studying (Elias, 2007). It can be inferred that individual traits affect individual attitudes, behaviors, and allocations of resources. Professional commitment, as their psychological or emotional connection to their future career, not only affects the shaping of individual psychological positive traits in different family environments but also acts as an individual trait to handle academic or professional pressure and exhibit good competence (Fors, 2016). Accordingly, the following are hypothesized:

Hypothesis 3: Professional commitment directly regulates the correlation between family environment and occupational identity.
Hypothesis 4: Professional commitment regulates the impact of the family environment on the psychological capital of publicly funded normal students. When professional commitment is high, the impact of the family environment on psychological capital is weak. When professional commitment is weak, the impact of the family environment on psychological capital is strong.
Hypothesis 5: Professional commitment has a moderating effect on the mediating role of psychological capital in the correlation between family environment and occupational identity. With high professional commitment, psychological capital exerts a stronger positive impact on occupational identity.

**Figure 1:**
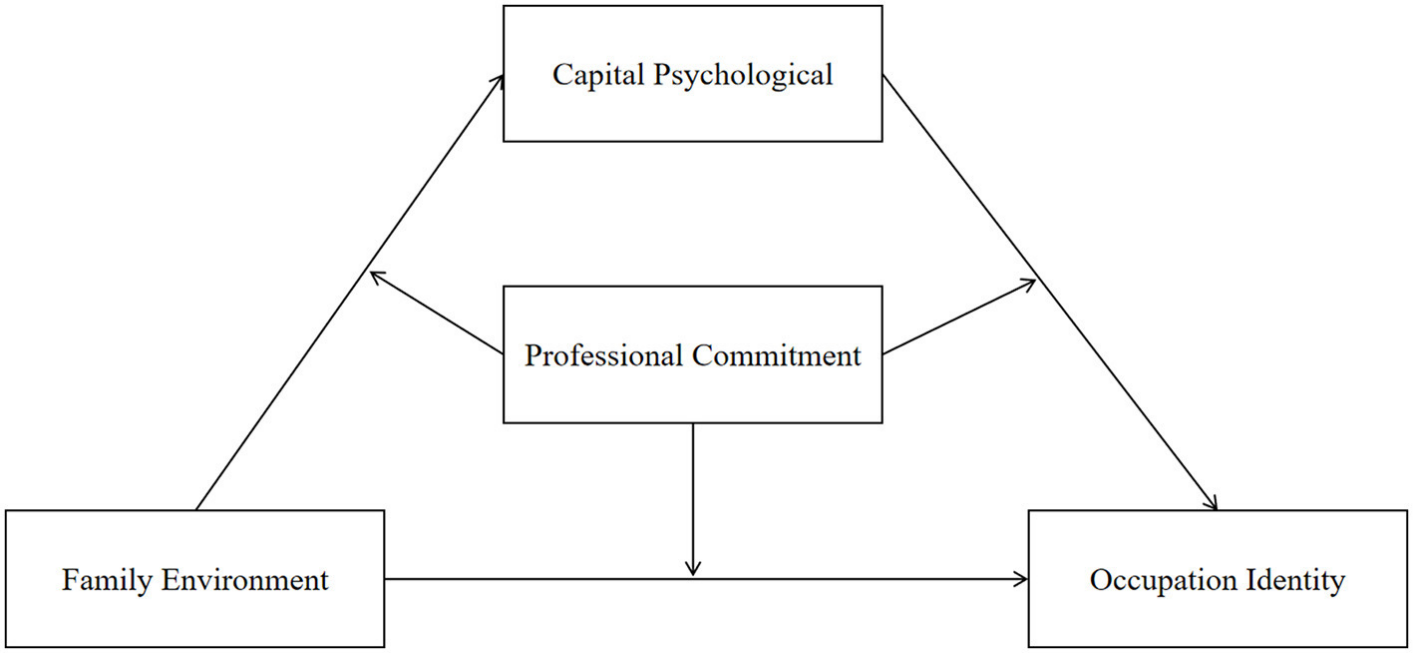
Theoretical model.

In summary, based on previous studies, we created and relied on the following theoretical model, as illustrated in Figure 1.

## 3. Methods

### 3.1. Samples and procedure

We selected freshmen to seniors from nine majors at seven normal universities in Sichuan to participate in the survey, which used cluster sampling and online questionnaire methods. The researchers randomly selected a class of publicly funded normal students from seven schools, contacted the class counselor to send the network questionnaire link to the entire class, asked each student to carefully complete the questionnaire, checked the completion time, and reminded students who did not answer in time. A total of 419 questionnaires were received. After excluding the questionnaires with outliers, 395 valid samples were obtained, and the sample effective rate was 94.3%.

Before data analysis, data cleaning is performed in accordance with the subsequently specified process and standards. First, data entry errors are checked, such as an extraneous 7 on the six-point Likert scale. Second, unusual cases are identified and removed, including missing data appearing in a non-random fashion, cases in which the same option is selected >50% of the time, missing data constituting >25% of the whole number of items, and the results in which the creative achievement *Z*-score is >3, which are identified as data anomalies or outliers. Afterward, 24 participants are excluded from the original sample after data cleaning. Consequently, 395 participants were chosen for the following data analysis process.

The demographic variables of the total sample were as follows (Table 1): 69 male students (17.5%) and 326 female students (82.5%); 98 only children (24.8%) and 297 non-only children (75.2%); and 32 (8.1%) from teachers' families and 363 (91.9%) from non-teachers' families. In addition, 11 (2.8%) students were from Chengdu, 172 (43.5%) from Mianyang, Deyang, and other cities and prefectures with the top 7 GDP in 2019, 68 (17.2%) from Liangshan, Neijiang, and other cities and prefectures with the top 8–13 GDP in 2019, and 142 (35.9%) from Panzhihua, Ziyang, and other cities and prefectures with the top 14–21 GDP in 2019, and 2 from outside Sichuan province (0.51%). Then, 136 students were freshmen (34.4%), 67 sophomores (17.0%), 131 juniors (33.2%), and 61 seniors (15.4%). To ensure the study's reliability and validity, the measurement questionnaires used were mature questionnaires that had been repeatedly used previously.

**Table 1:** Distribution table of demographic variables.

**Variable**	**Classification**	**Quantity**	**Proportion (%)**
Gender	Male	69	17.5
	Female	326	82.5
Only children or not	Only children	98	24.8
	Non-only children	297	75.2
From teacher family or not	Teacher family	32	8.1
	Non-teacher family	363	91.9
Cities	Chengdu	11	2.8
	The top 7 GDP cities	172	43.5
	The top 8–13 GDP cities	68	17.2
	The top 14–21 GDP	142	35.9
	Cities outside Sichuan province	2	0.51
Grade	Freshmen	136	34.4
	Sophomores	67	17.0
	Juniors	131	33.2
	Seniors	61	15.4
Total		395	100

### 3.2. Instruments

#### 3.2.1. Occupational identity scale

The occupational identity scale for publicly funded normal students compiled by Zhao Hongyu is categorized into three dimensions, namely, internal value identity, external value identity, and will behavior identity, with a total of 15 questions. Internal value identification denotes the value judgment of inherent work attributes, such as work content and mode. External value identification denotes the value judgment of professional social attributes, such as the working environment and social status. Will behavior identity denotes the behavior tendency driven by internal and external value identities (Zhao et al., 2012). The scale adopts the four-point scoring method, where one point represents “completely inconsistent” and four points represent “very consistent.” The higher the score, the higher the sense of identity. In this study, the internal consistency reliability of the scale was 0.924.

#### 3.2.2. Psychological capital scale

The psychological capital scale developed by Luthans and revised by Song Hongfeng for Chinese college students (Psychological Capital Questionnaire, PCQ) is categorized into four dimensions, namely, self-efficacy, hope, optimism, and tenacity, with a total of 16 questions (Song and Mao, 2012). The scale adopts the five-point scoring method, where one point represents “complete non-compliance” and five points represent “complete compliance.” The higher the score, the more positive the psychological state. In this study, the internal consistency reliability of the scale was 0.876.

#### 3.2.3. Professional commitment scale

The scale for college students' professional commitment compiled by Lian et al. is divided into four dimensions, namely, emotional commitment, ideal commitment, normative commitment, and continuous commitment, with a total of 27 questions (Lian et al., 2005). The scale adopts the five-point scoring method, where one score represents “complete non-compliance,” and five scores represent “complete compliance.” The higher the score, the higher the degree of commitment to the major. In this study, the internal consistency reliability of the scale was 0.863.

#### 3.2.4. Family environment scale

The family environment scale was compiled by Moss et al. and translated and revised by Fei in 1991. The scale has 10 subscales. In this study, three scales of intimacy, culture, and control with high reliability were selected. The answer “no” is scored 0, while the answer “yes” is scored 1. The higher the score on the intimacy dimension, the higher the degree of help and support among family members. The higher the score on the cultural dimension, the higher the degree of family participation in cultural activities. Moreover, the higher the score on the control dimension, the stronger the fixed family rules and procedures representing the family environment. In this study, the internal consistency reliability of the intimacy scale was 0.536, the culture scale was 0.510, and the control scale was 0.750.

### 3.3. Data analysis

We analyzed the data in four steps. First, we performed the common method deviation analysis, descriptive statistical analysis, correlation analysis, independent-sample *t*-test, and one-way ANOVA using SPSS 22. Second, we conducted four confirmatory factor analyses (CFA) and reliability analyses to measure the construct validity and reliability of the scales. Then, we created a structural equation model (SEM) with a second-order stereotype latent variable to confirm our theoretical model. Third, using the non-parametric percentile bootstrap method with deviation correction, we tested the mediating effect of psychological capital. Fourth, the latent regulatory structural equation was used to fit the models of independent variables, dependent variables, intermediary variables, and regulatory variables. Of note, all CFA and SEM analyses were conducted with Mplus 7.1. The models were tested using maximum-likelihood estimation of the sample covariance matrix.

## 4. Results

### 4.1. Common method deviation analysis

In the research design procedure, the questionnaire was arranged randomly, and some items used reverse questions and anonymous questionnaires to control the possible homologous variance. After data collection, Harman used the single-factor analysis test method of integrating all measurement topics into one variable and used the principal component analysis method to extract 21 factors with characteristic roots >1, with a cumulative interpretation of 63.339%, of which the first principal component factor explained 19.161% of the variation, which was < 50% of the variation interpretation. The data showed that the common method deviation did not exert a significant impact on the conclusion.

### 4.2. CFA and reliability

As suggested by Fornell and Larcker's suggestions, standardized factor load coefficients, composite reliability, convergent validity, and discriminant validity were used to test the model's reliability and validity. In this study, all CFA and SEM analyses were conducted using Mplus 7.1. Moreover, the models were tested using maximum-likelihood estimation of the sample covariance matrix. If the factor load is >0.7, the constituent reliability is >0.7, and the convergent validity is >0.5, the model is considered to have good validity, and if the square root of convergent validity is greater than the correlation coefficient of each latent variable, it reveals that the measurement model has good discrimination.

The occupational identity scale includes three dimensions, namely, internal value identity, external value identity, and will behavior identity. The fitting indexes of the second-order model were as follows: χ^2^/df = 1.95, CFI/TLI = 1.01, RMSEA = 0.049, and SRMR = 0.035. The standardized factor load of each observed variable was >0.7. In addition, the combined reliability of the three dimensions was 0.912, 0.825, and 0.798, respectively, which were greater than the recommended 0.7. Furthermore, the convergent validity was 0.723, 0.612, and 0.597, respectively, which were greater than the recommended 0.5. Hence, the model had good convergence.

The psychological capital scale includes four dimensions, namely, self-efficacy, hope, optimism, and tenacity. The fitting indexes of the second-order model were as follows: χ^2^/df = 3.8, CFI/TLI = 1.01, RMSEA = 0.086, and SRMR = 0.043, which fulfilled the recommended reference values. The standardized factor load of each observed variable was >0.7. The combined reliability of the four dimensions was 0.707, 0.754, 0.747, and 0.797, respectively, —all greater than the recommended 0.7. Moreover, the convergent validity was 0.546, 0.535, 0.598, and 0.568, respectively, which were greater than the recommended 0.5. Hence, the model had good convergence.

The professional commitment scale includes four dimensions, namely, emotional commitment, ideal commitment, normative commitment, and continuing commitment. The fitting indicators of the second-order model were as follows: χ^2^/df = 3.4, CFI/TLI = 1.02, RMSEA = 0.079, and SRMR = 0.052, which fulfilled the recommended reference values. The standardized factor load of each observed variable was >0.7, and the combined reliability of the four dimensions was 0.780, 0.804, 0.831, and 0.747, respectively, all greater than the recommended 0.7. Furthermore, the convergent validity was 0.516, 0.506, 0.555, and 0.582, respectively, which were greater than the recommended 0.5. Hence, the model had good convergence.

The family environment scale includes three dimensions, namely, intimacy, culture, and control. The fitting indexes of the second-order model were as follows in Table 1: χ^2^/df = 1.41, CFI/TLI = 1.01, RMSEA = 0.032, and SRMR = 0.039, which fulfilled the recommended reference values. The standardized factor load of each observed variable was >0.7, the combined reliability of the three dimensions was 0.726, 0.705, and 0.746, respectively, which were greater than the recommended 0.7, and the convergent validity was 0.599, 0.542, and 0.588, all greater than the recommended 0.5. Hence, the model had good convergence.

Tables 2, 3 present a summary of the CFA item indices, reliability (internal consistency), and factor loadings for the stereotype model.

**Table 2:** Goodness-of-fit indices and reliability of scales.

**Scale**	**χ^2^/df**	**CFI/TLI**	**RMSEA**	**SRMR**
Occupational identity	1.95	1.01	0.049	0.035
Psychological capitals	3.8	1.01	0.086	0.043
Professional commitment	3.4	1.02	0.079	0.052
Family environment	1.41	1.01	0.032	0.039

RMSEA, root-mean-square error of approximation; SRMR, standardized root-mean-square residual; CFI, comparative fit index; TLI, Tucker–Lewis index.

**Table 3:** Reliability and discriminant validity of each subscale.

	**CR**	**AVE**	**1**	**2**	**3**	**4**	**5**	**6**	**7**	**8**	**9**	**10**	**11**	**12**	**13**	**14**
1. Intrinsic value	0.912	0.723	0.850													
2. External value	0.825	0.612	0.753	0.782												
3. Will behavior	0.798	0.597	0.728	0.708	0.773											
4. Self-efficacy	0.707	0.546	0.315	0.166	0.387	0.739										
5. Hope	0.754	0.535	0.389	0.327	0.396	0.688	0.731									
6. Optimistic	0.747	0.598	0.389	0.391	0.405	0.534	0.764	0.773								
7. Tenacity	0.797	0.568	0.446	0.392	0.457	0.470	0.591	0.705	0.754							
8. Emotional	0.780	0.516	0.587	0.412	0.585	0.512	0.772	0.662	0.596	0.718						
9. Ideal	0.804	0.506	0.551	0.452	0.670	0.504	0.649	0.588	0.613	1.008	0.711					
10. Normative	0.831	0.555	0.389	0.160	0.243	0.304	0.402	0.332	0.384	0.657	0.559	0.745				
11. Continuation	0.747	0.582	0.553	0.390	0.563	0.274	0.366	0.485	0.523	0.826	0.822	0.816	0.763			
12. Intimacy	0.726	0.599	−0.291	−0.296	−0.252	−0.122	−0.233	−0.287	−0.303	−0.366	−0.278	−0.226	−0.295	0.774		
13. Culture	0.705	0.542	−0.169	−0.186	−0.295	−0.164	−0.299	−0.350	−0.194	−0.238	−0.247	−0.030	−0.074	0.356	0.736	
14. Controllability	0.746	0.588	−0.051	−0.066	−0.192	−0.118	−0.201	−0.300	−0.141	−0.144	−0.217	−0.009	−0.202	0.216	0.331	0.767

The items on the diagonal represent the square roots of the AVE; off-diagonal elements are the correlation estimates.

### 4.3. Descriptive statistics and correlation analysis

We used SPSS 22.0 to statistically analyze the mean, standard deviation, and correlation of each variable (Table 4). According to Table 4, the average of the overall level of family environment was 0.528, which was at the medium level, suggesting that the overall family environment of publicly funded normal students was relatively general. The average scores of occupational identity and psychological capital were 3.18 and 3.80, respectively, suggesting that the overall level of psychological capital and the sense of identity with teachers' profession of publicly funded normal students were at the upper-middle level, while the average value of professional commitment was 4.92, suggesting that publicly funded normal students showed love and recognition for their major, as well as a good attitude and positive behavior.

**Table 4:** Summary of intercorrelations, means, and standard deviations for scores on subscales.

	**M**	**SD**	**1**	**2**	**3**	**4**
1. Family environment	0.528	0.116	1			
2. Occupational identity	3.180	0.463	−0.104^*^	1		
3. Psychological capital	3.800	0.414	−0.179^**^	0.443^***^	1	
4. Professional commitment	4.920	0.483	−0.236^**^	0.507^**^	0.618^**^	1

*N* = 335.

^*^P < 0.05.

^**^P < 0.01.

^***^P < 0.001.

### 4.4. *t*-test and one-way ANOVA analysis

In addition, the independent sample *t*-test showed that there was no statistically significant gender difference in occupational identity (*t* = 0.009, *P* > 0.05), no statistically significant difference in whether students came from teachers' families (*t* = −0.760, *P* > 0.05), and no statistically significant difference in whether they were an only child (*t* = −0.490, *P* > 0.05).

The one-way ANOVA showed a significant difference between freshmen and juniors (*F* = 4.443, *P* < 0.05), and juniors' occupational identity was significantly higher than that of freshmen. From the perspective of students' origin, no significant difference was found in occupational identity (*F* = 0.958, *P* > 0.05), that is, no statistically significant difference was found in occupational identity between publicly funded normal students from areas with high or low economic ranking.

### 4.5. A test of the mediating effect of psychological capital

Before the intermediary effect test, the measurement model without the intermediary effect was tested. The results revealed that the measurement model fitted well [χ^2^/df < 2, RMSEA = 0.047, 90% confidence interval (CI): (0.044–0.050), CFI = 0.879, TLI = 0.872, SRMR = 0.056]. Per the mediating effect test based on the structural equation proposed by Wen et al., the first step was to build an SEM from independent variables to dependent variables. The second step was to build an SEM incorporating intermediate variables to attain the CI based on the bootstrap method. As the sampling distribution of the mediating effect AB often does not obey the normal distribution, the non-parametric percentile bootstrap method with deviation correction was used to estimate the CI of the mediating effect to attain the robust standard error CI of parameter estimation. If the 95% CI does not contain zero, it means that the statistics are significant.

The first step was to test the direct effect of family environment on occupational identity. The results showed that the model fitted well [χ^2^ = 2,176.563, CFI = 0.878, TLI = 0.870, RMSEA = 0.047, 90% CI (0.044–0.050), SRMR = 0.059]. After controlling the variables, such as gender, whether they come from the teachers' families, whether they were the only child, grade, and place of origin, the family environment exerted a significant predictive impact on occupational identity (β= −0.152, *P* < 0.001), and the explanation for the variation of occupational identity was 23.9%. Hence, hypothesis 1 is confirmed.

**Figure 2:**
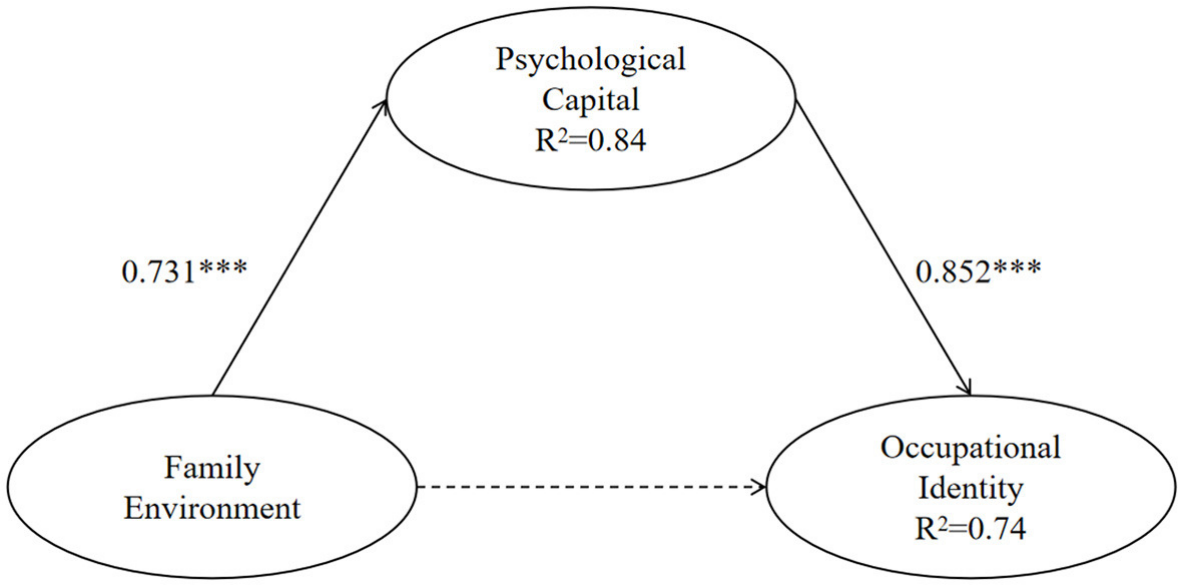
The mediating effect of psychological capital. ^*^P < 0.05, ^**^P < 0.01, ^***^P < 0.001.

The second step was to add psychological capital as a mediating variable based on the original model. Figure 2 illustrates the mediating effect of psychological capital. The results showed that the model fitted well [χ^2^ = 2,160.336, CFI = 0.877, TLI = 0.870, RMSEA = 0.047, 90% CI (0.044–0.050), SRMR = 0.057]. The variation explanations of psychological capital and occupational identity were divided into 83.8 and 73.9% (β = 0.731, *P* < 0.001). Psychological capital exerted a significant predictive effect on occupational identity (β = 0.852, *P* < 0.001). The prediction of family environment on occupational identity was not significant (β = 0.07, *P* > 0.05). In addition, the mediating effect of psychological capital between family environment and occupational identity was established [95% CI (1.343–2.755)]. Thus, psychological capital was found to play a complete mediating role in the correlation between family environment and occupational identity. Hence, hypothesis 2 is confirmed.

### 4.6. The moderation effect of professional commitment

According to Fang and Wen (2018), the fitting test of the independent variable, dependent variable, intermediate variable, and regulatory variable models was conducted using latent modulated structural equations (LMS). In the test of the latent regulation effect, the traditional regression model does not consider the measurement error of the index, which generally distorts the parameter estimation results. However, LMS has unique advantages and does not need to construct the product index, avoiding the problem of inconsistent parameter estimation due to the generation of different product indexes. Also, it does not require the interaction term to obey the normal distribution, thus avoiding the estimation deviation due to the non-normal distribution of the product term. As suggested by Maslowsky et al. (2015), first, the main effect of professional commitment was added per the intermediary model. The results revealed that β = −0.228, *P* > 0.05, and the direct moderation effect is not significant. Hence, hypothesis 3 is not confirmed. According to Muller et al. (2005), if the direct path is not regulated, a regulated mediation model is established.

The second step was to establish the benchmark model M0 without potential interaction terms. The results revealed that the model fitted well [χ^2^ = 2,181.455, *P* < 0.001, CFI = 0.877, TLI = 0.869, AIC = 29,849.424, RMSEA = 0.047, 90% CI of RMSEA (0.044–0.050), and SRMR = 0.057].

The third step was to establish and add latent regulation (interaction) item 1 (family environment) × professional commitment) and potential regulation (interaction) item 2 (psychological capital) × regulated mediation model M_1_ of professional commitment. The results showed that AIC = 29,849.329 and the AIC (29,849.424) value remained unchanged compared with the benchmark model M0, suggesting that the adjusted SEM model M_0_ has not deteriorated compared with the benchmark SEM model. Meanwhile, the log-likelihood ratio test was used to compare the H_0_ value in the benchmark model and calculate the −2LL value (the difference between the likelihood ratio of the basic model and the adjusted intermediary model). The log-likelihood of the mediation model with adjustment was −14,755.344, which increased by 0.368 compared with the log-likelihood value (−14,755.712) of the benchmark model, that is, −2LL= 0.368; the difference value of the degree of freedom of the model parameters was 1, and the χ^2^ test of the −2LL value was significant (*P* < 0.05). The mediation model with regulation was better than the benchmark model. Thus, professional commitment exerted no significant moderating effect on family environment and psychological capital (β = −0.119, *P* > 0.05). Furthermore, professional commitment exerted a significant moderating effect on the correlation between psychological capital and occupational identity (β = 0.077, *P* < 0.001). Hence, hypothesis 4 is not confirmed, while hypothesis 5 is confirmed.

**Figure 3:**
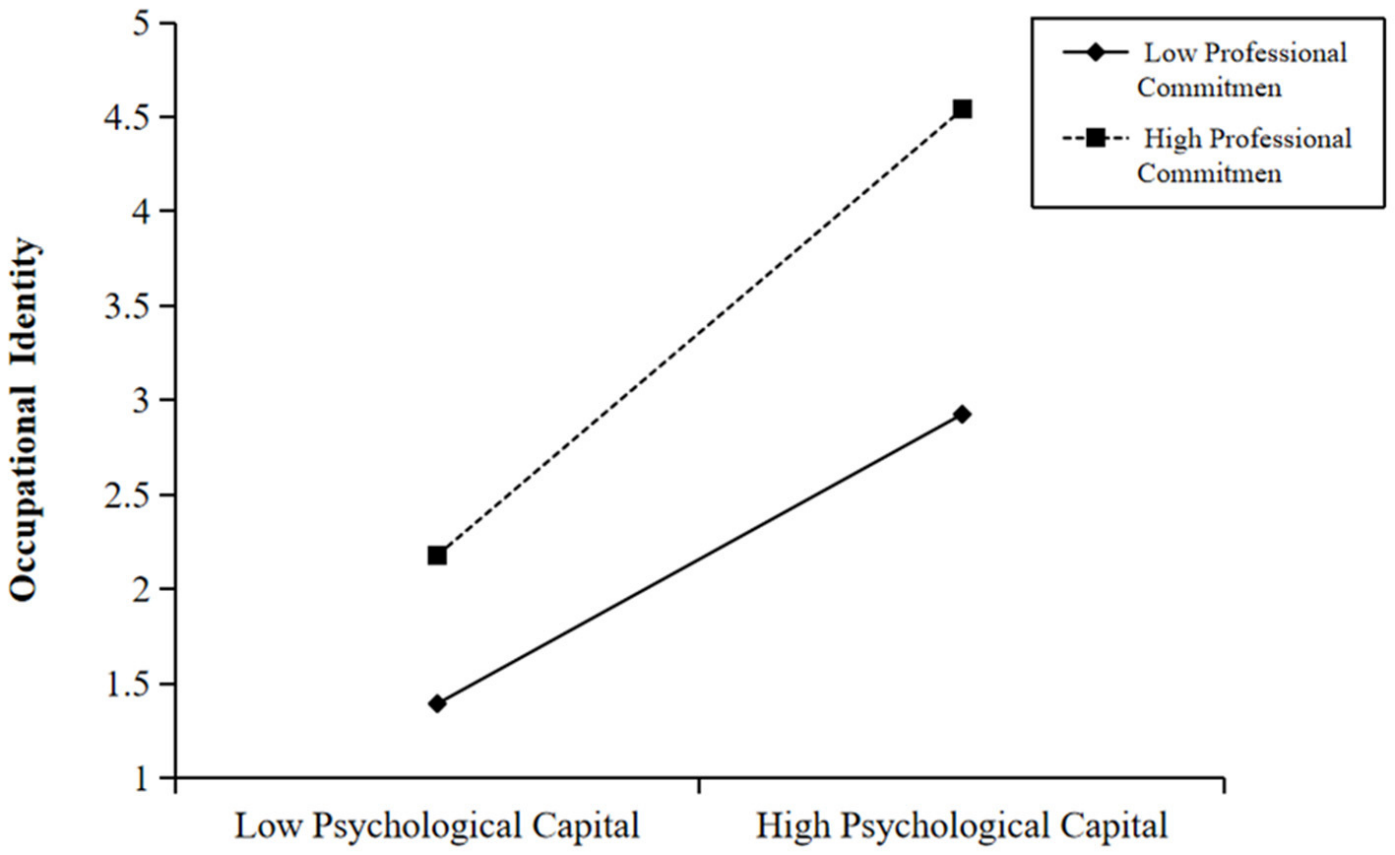
The moderation effect of professional commitment.

To more clearly reveal the correlation between psychological capital and the interaction effect of professional commitment, a simple slope test was conducted to test the mechanism of regulatory effect based on the standard deviation of professional commitment above and below the mean. Drawing a simple interaction effect analysis chart demonstrated that the intermediary process of family environment affecting occupational identity through psychological capital was regulated by professional commitment (Figure 3). In addition, professional commitment played a positive regulatory role between psychological capital and occupational identity. For publicly funded normal students with high professional commitment, the positive impact of psychological capital on occupation identity was found to be stronger.

## 5. Discussion

The occupational identity of publicly funded normal students influences the quality of normal students' training and the cultivation of rural teachers. Taking the publicly funded normal students in local normal universities as the research object, this study investigated the mediating and moderating effect of the family environment of publicly funded normal students on occupational identity, as well as examined the combined effect of psychological capital and professional commitment variables. The findings elucidated how the family environment of normal students affects their occupational identity. The model demonstrates that environmental factors affect the internal value judgment of publicly funded normal students and then affect individual occupational identity.

### 5.1. Characteristics of the occupational identity of publicly funded normal students

The results revealed that publicly funded normal students' occupational identity as teachers is at a medium level, and they show a good attitude and positive level toward their major. In addition, we explored the correlation between demographic variables and the occupational identity of publicly funded normal students. The findings revealed that gender exerts no significant impact on occupational identity. Nevertheless, the difference in the loss of teachers' gender is a matter of great concern. Previous studies claimed that the loss rate of female teachers is lower because of their traditional role orientation, while the loss rate and career change rate of male teachers are higher (Chan et al., 2008). This study confirms no difference in job identity between student teachers in terms of gender. The reason for the high turnover rate of male teachers could be more due to the career development issues and challenges faced after service. Second, whether an individual is an only child, whether he comes from a teacher's family, and different student sources are not the main factors affecting the occupational identity of normal students. Significant differences exist in occupational identity between juniors and freshmen. With the increase in grades, the occupational identity of normal students revealed an upward trend, suggesting that normal education in the university plays a positive guiding role in the cultivation of students' occupational identity attitudes. With the learning of normal courses and the perception of internships in educational practice, students can further feel a sense of value and happiness as teachers, enhancing their occupational identity.

### 5.2. Psychological capital is totally mediated by the correlation between family environment and occupational identity

This study verifies that the family environment exerts a positive predictive impact on occupational identity, which is completely mediated by psychological capital. The higher the psychological capital of publicly funded normal students with a good family environment, the higher the sense of occupational identity. This result not only validates the previous research but also draws new conclusions.

On the one hand, the family environment exerts a positive impact on occupational identity. The family environment with a high degree of intimacy, rich culture, and low control positively correlates with the occupational identity of publicly funded normal students, which can be explained by psychological capital. The parent–child attachment relationship and personality quality attained by the family are similar to the “habit” proposed by Bourdieu, which imperceptibly shapes psychological capital and promotes the recognition of the teachers' profession (Bourdieu and Wacquant, 1998). In addition, a good family environment leads to a good attachment relationship between parents and children, which can be elucidated by psychological capital. In addition, good parent–child attachment helps to improve teenagers' psychological capital, form good self-awareness, and develop a more positive explanation of others and events (Nickerson and Nagle, 2004); this internal psychological demand can be interpreted as psychological capital “positive evaluation of the environment and good expectation of the possibility of success, which is based on the effort and persistence toward the goal” (Luthans et al., 2007a,b). Reportedly, resilience, hope, and optimism correlate with employee satisfaction and job happiness. When individuals are in a satisfied and pleasant psychological state, they are more likely to have higher recognition of their profession or occupation. A good family environment nurtures teenagers' positive emotions. Individuals have tenacity, hope, and optimism, which usually results in teenagers being full of self-confidence and positive optimism. This makes it simpler for them to attain satisfaction after success, enabling them to treat their decisions more actively.

On the other hand, the psychological capital of publicly funded normal students is totally mediated by the correlation between family environment and occupational identity, which helps to elucidate the formation factors of publicly funded normal students' occupational identity, further deepening the understanding of the correlation between family environment and occupational identity. It has been considered that adults after work have high psychological capital, which usually makes individuals confident, optimistic, overcome difficulties, strive hard, exhibit positive emotions, attain a sense of accomplishment after success, and then bring a sense of satisfaction, making them more willing to work in the organization (Jiao and Zheng, 2019). However, as student teachers, publicly funded normal students have not yet entered the field of work. Is this group's occupational identity also related to psychological capital? This study confirms that psychological capital factors exert a significant impact on the occupation identity of publicly funded normal students. Although the orientation factors of publicly funded normal students differ from ordinary normal students and their cognition of teachers' careers could be vague, in the next few years of college life, when we really learn and understand the characteristics and requirements of the teaching industry from the theoretical and practical levels, a large part of their expectations for the teacher industry and their evaluation of whether they are suitable for the teacher industry still stems from psychological capital factors. Besides, the shaping of psychological capital is not achieved overnight. A good family environment promotes the improvement of publicly funded normal teachers' psychological capital, and a higher psychological capital can augment the occupational identity of the teaching industry.

### 5.3. Professional commitment plays a positive regulatory role between psychological capital and occupational identity

This study establishes that good professional commitment plays a positive role in promoting the occupational identity of publicly funded normal students. Previous studies explored the impact of teacher support and teaching efficacy on occupational identity, but this study investigates the impact of another variable—professional commitment.

The SEM showed that the direct regulatory effect of professional commitment on family environment and occupational identity was not significant. In addition, professional commitment was found to play a regulatory role in the intermediary process of “family environment psychological capital occupational identity,” which is manifested in that the second half of the intermediary chain is regulated by professional commitment.

Specifically, the interaction between professional commitment and psychological capital exerts a significant positive predictive impact on occupational identity. Professional commitment might enhance occupational identity by enhancing psychological capital. Together with the results of relevant analyses, professional commitment is conducive to the improvement of occupational identity. With the enhancement of professional commitment, the impact of the family environment tends to weaken, perhaps because when individuals start college life, the emotional and ideal source of their teachers' industry has gradually shifted from family to the educational concept of the university and the influence of teachers and peers. Their cognition and recognition of the teaching industry come from their psychological potential and positive advantages. Even if they have a vague understanding of the teaching industry when filling out the college entrance examination, they can also complete the internal behavior of teacher role recognition through psychological capital. Second, professional commitment does not exhibit a regulatory effect on the path of the first half of the intermediary process (family environment psychological capital), demonstrating that the shaping of individuals by the family environment is primarily to enhance their psychological capital and cannot directly affect their love for their major; however, good psychological capital is still conducive to publicly funded normal students to develop the concept and behavior of teachers' occupational identity.

## 6. Conclusion

The association between occupational identity and the family environment of publicly funded students at local normal colleges is multifaceted and complex. Through a survey of 395 Chinese publicly funded normal students in seven local normal universities in Sichuan province, this research article examined the correlation among occupational identity, psychological capital, professional commitment, and family environment. The study findings indicate that the family environment plays a significant role in the professional identification of publicly funded normal school students as rural teachers. Although the family environment does not directly shape the occupational identity of publicly funded normal students who are about to return to rural areas in their hometowns, conversely, the proposed influence indirectly affects their career identification by stimulating their professional effect, thereby reinforcing their belief in becoming a future rural teacher. In addition to this, the mediating role of psychological capital in the relationship between family environment and occupational identity, as well as the moderating impact of professional commitment, can support counselors and educators in devising intervention measures to enhance students' occupational identity.

From this perspective, the research findings of this article also hold important practical significance. Thus, there is potential for much more to be done to help the students. First, educators, students, and parents should be aware that the family and school play a crucial role in promoting the professional identity of publicly funded normal students. In particular, students are prominently influenced by their families when choosing majors. Since students may have insufficient preparation to comprehend the possible challenges in the hardship areas, there is a need to improve their rational and emotional understanding of rural education, strengthen guidance on rural teacher career planning, and assist them with explicit professional goals. Second, it is essential to track the professional development of students for several years for dynamic monitoring of changes in professional identity. Accordingly, normal universities may need to take more responsibility for professional commitment in the case of students' families where parental support is insufficient, while simultaneously encouraging their families to give them more emotional support. Finally, the researchers of this study expect that this study will help future teachers who are about to commence their careers in difficult fields by better preparing them from the perspective of families and universities and giving them the confidence to become rural teachers.

## 7. Limitations and future research direction

Owing to certain limitations, the research findings of this study should be interpreted with caution. First, this article used cross-sectional data; therefore, the causal relationship between variables cannot be accurately inferred from this study. Hence, the adoption of time lag analysis and experimental methods can be considered in future research to perform a more profound discussion on the causal association between these variables. Second, this study was carried out at normal universities in Sichuan province within a specific context and the culture of China, which may be different if carried out in other regions. The country's setting is a factor that considerably influences the results of a study (Bergeron et al., 2014). While we consider the contextual factors, the results may be more interesting if the influence of cultural values on such study variables were to be examined empirically.

## Data availability statement

The raw data supporting the conclusions of this article will be made available by the authors, without undue reservation.

## Ethics statement

Ethical review and approval was not required for the study on human participants in accordance with the local legislation and institutional requirements. Written informed consent from the patients/participants or patients/participants legal guardian/next of kin was not required to participate in this study in accordance with the national legislation and the institutional requirements.

## Author contributions

JZ: conceptualization, data collection, and writing the manuscript. YL: investigation, data analysis, and writing—reviewing visualization. ZM: reviewing and editing. BX: revised the manuscript. All authors discussed the results and contributed to the final manuscript.
